# Yellow Fever in the South

**Published:** 1897-10

**Authors:** 


### YELLOW FEVER IN THE SOUTH.

IT IS about a month since the first cases of yellow fever appeared at Ocean Springs, Miss. Since that time the disease has spread to New Orleans and Mobile, and four cases, real or suspected, have been reported as far north as Cairo, Ill. Up to the present writing (September 24th) about 250 cases of genuine yellow fever have been officially reported at the office of the surgeon-general of the marine hospital service at Washington. It is estimated that about 100 suspected cases have fallen under the cognisance of the local health boards principally in the states of Alabama, Mississippi and Louisiana.

While the aggregate number of cases appears somewhat formidable and the recent spreading of the infection appears to have excited apprehension in the localities invaded, it is gratifying to know that the number of deaths announced is estimated at less than 5 per cent. of the whole number treated. Moreover, it is probable that the death-rate will diminish rather than increase under the influence of cooler weather, that has prevailed during the past week. As the season of frost approaches, too, apprehension will be lessened and confidence will be established. As compared with the epidemic in Florida in 1888, when the percentage of deaths was more than double the present invasion, and as further compared with earlier epidemics during which the mortality was at least 20 per cent. of the whole number of cases, the present outbreak may be regarded as phenomenally mild. This speaks well for the sanitary regulations enforced by the general and local governments.

While, therefore, it is gratifying to contemplate that great loss of life is not to be feared from the present epidemic, it cannot be denied that the business interests of the south are suffering to a considerable degree, resultant from the necessary interference with traffic and commerce and the establishment of rigid quarantine regulations. Furthermore, the suspicion cannot be resisted that the state boards of health in the Gulf region have not been as vigilant in preventing the entrance of persons suffering from yellow fever into their borders as they should have been. Let us hope that the lesson taught will be a profitable one in the future.

